# Protective Effect of a Highly Enriched Nacre-Derived Neutral Polysaccharide Fraction on D-Galactose-Induced Pancreatic Dysfunction

**DOI:** 10.3390/molecules30173555

**Published:** 2025-08-30

**Authors:** Heng Zhang, Yasushi Hasegawa

**Affiliations:** College of Environmental Technology, Muroran Institute of Technology, 27-1 Mizumoto, Muroran 050-8585, Japan; 24096004@muroran-it.ac.jp

**Keywords:** nacre extract, aging, pancreas, insulin, polysaccharide

## Abstract

Nacre, the iridescent inner layer of mollusk shells, has long been traditionally used in medicine. While we have previously demonstrated its anti-aging effects on muscle and skin, its impact on pancreatic dysfunction and glucose metabolism remains unclear. In this study, we aimed to isolate and identify an active component from nacre extract that improves glucose metabolism and to evaluate its potential to prevent or ameliorate pancreatic dysfunction and glucose metabolic abnormalities in a D-galactose-induced aging mouse model. A polysaccharide component was successfully isolated using a combination of reverse-phase and ion-exchange chromatography. Structural analyses revealed that it was primarily composed of glucose, mannose, and rhamnose, which together accounted for approximately 87% of the total monosaccharide content. Further characterization by FT-IR spectroscopy and MALDI-TOF-MS confirmed its identity as a neutral polysaccharide with glycosidic linkages and an estimated molecular weight of approximately 5000 Da. Intraperitoneal administration of this polysaccharide significantly improved glucose tolerance and prevented a decline in serum insulin levels in D-galactose-induced aging mice. Immunohistochemical analysis of pancreatic tissues revealed that the polysaccharide preserved insulin expression and suppressed the D-galactose-induced upregulation of cellular senescence and apoptosis markers. These findings suggest that this nacre-derived polysaccharide effectively mitigates pancreatic dysfunction and glucose metabolic dysfunction, indicating its potential as a natural therapeutic agent for age-related metabolic disorders.

## 1. Introduction

Aging is associated with a progressive decline in glucose homeostasis, increasing the risk of metabolic disorders such as type 2 diabetes and impaired glucose tolerance [1,2,3,4]. One major contributor to this decline is pancreatic dysfunction, which disrupts both insulin secretion and glucose regulation [5,6,7]. The pancreas consists of two functionally distinct compartments: endocrine cells, located in the islets of Langerhans, which regulate blood glucose levels through hormone secretion; and exocrine cells, which constitute the majority of the pancreatic tissue and are responsible for the production of digestive enzymes.

Among the endocrine cells, insulin-producing β-cells play a central role in maintaining glucose balance. However, aging impairs β-cell function through mechanisms such as cellular senescence, oxidative stress, apoptotic cell loss, and diminished regenerative capacity, ultimately resulting in decreased insulin production and impaired glucose regulation [7,8,9]. This dysfunction contributes to impaired glucose tolerance. Thus, maintaining pancreatic health is crucial for preventing age-related metabolic diseases.

To study aging-related metabolic dysfunction in vivo, the D-galactose-induced aging mouse model is widely used and well validated [10,11,12]. Chronic D-galactose administration mimics natural aging by inducing oxidative stress, inflammation, and cellular senescence [13,14]. It also produces histological alterations in pancreatic tissue that resemble those seen in physiological aging [15,16,17]. While D-galactose models have been extensively used to study brain, liver, and kidney aging, studies specifically focusing on pancreatic dysfunction and its effect on glucose metabolism remain limited [18,19,20]. Furthermore, D-galactose administration has been reported to impair glucose metabolism and reduce insulin secretion. In recent years, interest has grown in natural substances with potential anti-aging effects, particularly those capable of targeting cellular senescence or preserving organ function [21,22,23].

Nacre, the inner iridescent layer of mollusk shells, has a long history of use in traditional medicine and has demonstrated various biological activities [24]. Our previous studies demonstrated that nacre extract attenuates aging-related changes in muscle and skin tissues in D-galactose-induced aging models [25]. However, its effects on pancreatic dysfunction and glucose regulation have not yet been fully elucidated. Although nacre extract exhibits various biological activities, the specific components responsible for its beneficial effects have not yet been fully identified or characterized. The identification and characterization of these active constituents is crucial for elucidating the mechanisms underlying their anti-aging properties.

Previous studies have reported that nacre-derived polysaccharides possess distinct structural features associated with biological activity. For example, Yamagami et al. (2021) isolated a sulfated polysaccharide from nacre (~750 kDa) rich in galactose, glucose, mannose, and uronic acid, demonstrating antioxidant and neuroprotective effects in scopolamine-induced memory impairment [26]. More recently, Wako et al. (2024) reported that sulfated polysaccharides from nacre suppress amyloid β deposition, neuroinflammation, and tau phosphorylation in a chronic scopolamine-induced Alzheimer’s model [27].

Given this background, the present study aimed to determine whether nacre extract can prevent or ameliorate pancreatic dysfunction and associated metabolic abnormalities in a D-galactose-induced aging mouse model. In addition, we sought to isolate and identify the specific fraction of the extract responsible for improving glucose metabolism, with the goal of advancing the scientific understanding and practical application of nacre-derived bioactive substances. By clarifying the impact of nacre extract and its active components on glucose metabolism and pancreatic function, this research may contribute to the development of natural therapeutic strategies for promoting healthy aging and metabolic health.

## 2. Results

**Figure 1:**
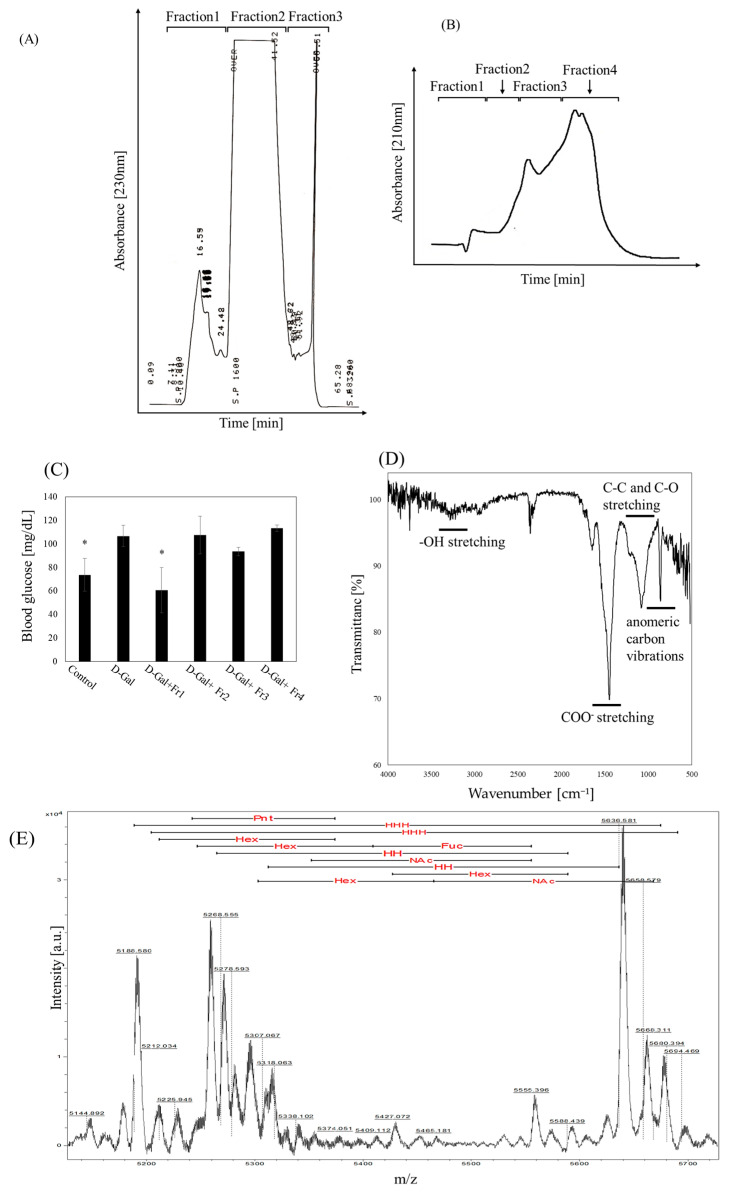
Fractionation of nacre extract and identification of fractions that attenuate D-galactose-induced elevation of blood glucose levels. (**A**) The nacre extract was separated into three fractions using a C18 reverse-phase column eluted with a linear gradient of 0–50% acetonitrile. (**B**) Fraction 3 obtained from the reverse-phase column was further separated using an ion-exchange column eluted with a linear gradient of 0–0.5 M NaCl. (**C**) Fasting blood glucose-lowering effect of each fraction obtained by ion-exchange chromatography. The experimental groups consisted of Control, D-galactose (D-Gal), D-Gal injected with Fraction 1 (Fr1), and groups receiving Fr2, Fr3, or Fr4. Data represents the mean ± standard deviation of four or five mice per group. * *p* < 0.05 vs. D-galactose group. (**D**) FT-IR spectrum of Fraction 1 (left panel) and (**E**) MALDI-TOF-MS spectrum of Fraction 1 (right panel). Fuc, fucose; Hex, hexose; Pnt, Pentose; NAc, N-acetyl hexosamine; HH, hexose–hexose; HHH, hexose–hexose–hexose.

D-galactose administration significantly increased fasting blood glucose levels in mice, confirming the induction of glucose metabolism abnormalities. To identify the component in nacre extract responsible for ameliorating these abnormalities, the extract was fractionated into four fractions using reverse-phase and ion-exchange chromatography (Figure 1A,B), and each fraction was evaluated for its effect on fasting blood glucose (Figure 1C). Among the four fractions, only Fraction 1 significantly suppressed the D-galactose-induced increase in fasting blood glucose (Figure 1C), while Fractions 2, 3, and 4 showed no such effect. This finding indicates that the administration of Fraction 1 can counteract the hyperglycemic effect caused by D-galactose, and suggests that the components responsible for improving glucose metabolism are concentrated in Fraction 1.

Biochemical analysis showed that Fraction 1 contained 3.9 mg/mL of carbohydrates and 0.1 mg/mL of protein (Figure 1C). Monosaccharide composition analysis revealed that Fraction 1 was predominantly composed of neutral sugars, with glucose, mannose, and rhamnose as the major components, collectively accounting for approximately 87% of the total sugar content (Table 1). Minor amounts of galactose, xylose, N-acetylgalactosamine, and other sugars were also detected. SDS-PAGE with silver staining showed no detectable protein bands (Supplementary Material), indicating negligible protein contamination. In addition, mass spectrometry yielded signals consistent with polysaccharide-derived components and did not reveal obvious non-polysaccharide peaks. These findings support the conclusion that Fraction 1 is predominantly a neutral polysaccharide with high purity.

FT-IR spectroscopy revealed spectral features characteristic of polysaccharides, including a broad O–H stretching band around 3300 cm^−1^, a C–H stretching peak near 2920 cm^−1^, and strong absorptions in the 1200–1000 cm^−1^ region corresponding to C–O, C–C, and glycosidic linkage. A prominent absorption near 1500 cm^−1^ was also observed, which may correspond to the amide II band or carboxylate groups (COO-) from trace amounts of peptide. Notably, absorption bands in the 900–700 cm^−1^ region, characteristic of anomeric carbon vibrations, further support the presence of polysaccharide structures [28,29,30]. MALDI-TOF mass spectrometry also confirmed the structural heterogeneity of Fraction 1 and showed that it is a polysaccharide containing hexose, pentose, fucose, and hexosamine residues. Major peaks near *m*/*z* 5000 suggest that the predominant components are polysaccharides with molecular weights around 5000 Da. Furthermore, ultrafiltration analysis also revealed that most of the bioactive substances in Fraction 1 had molecular weights below 10,000 Da, although high molecular weight polysaccharides exceeding 50,000 Da were also present in small amounts. Collectively, these results indicate that Fraction 1 consists mainly of neutral polysaccharides rich in glucose, mannose, and rhamnose, with a small amount of protein.

**Figure 2:**
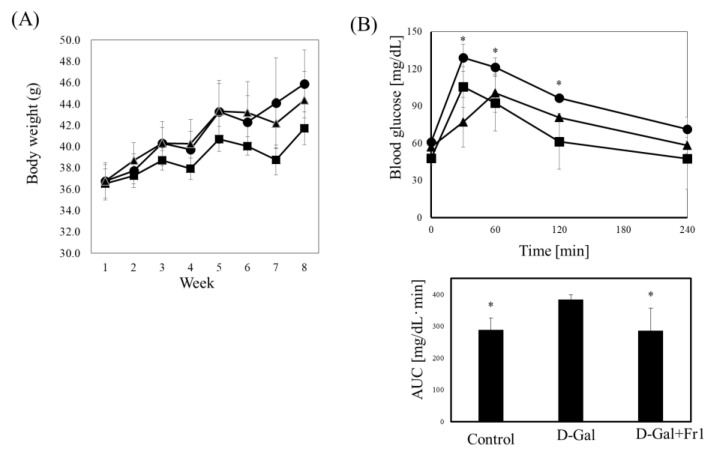

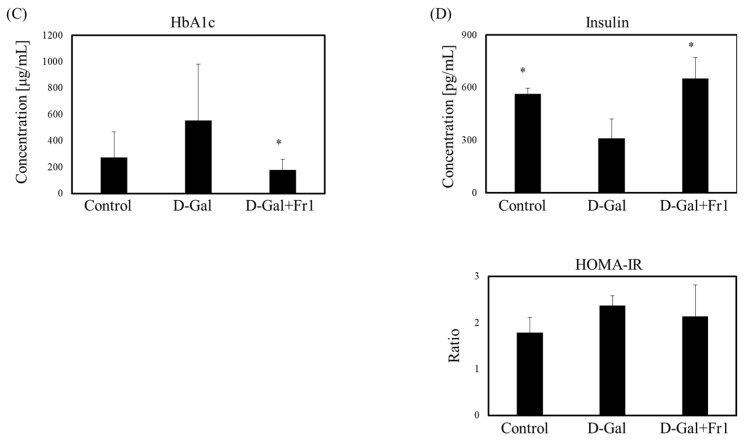
Effect of Fraction 1 on D-galactose-induced glucose dysfunction. (**A**) Body weight changes in the control (triangle), D-galactose (circle), and Fraction 1 (square) groups. (**B**) Glucose tolerance test results for the control (triangle), D-galactose (circle), and Fraction 1 (square) groups. After overnight fasting, glucose (2.0 g/kg) was orally administered, and blood glucose levels were measured at 0, 30, 60, 120, and 240 min (left panel). Area under the curve (AUC) values were calculated for each group (right panel). (**C**) Blood HbA1c levels quantified using an ELISA kit. (**D**) Insulin levels measured by ELISA, and HOMA-IR values calculated as: fasting blood glucose (mg/dL) × fasting insulin (μU/mL)/405. Data represents the mean ± standard deviation of five mice per group. * *p* < 0.05 vs. D-galactose group.

Daily intraperitoneal administration of D-galactose suppressed body weight gain in mice compared to the control group (Figure 2A). However, co-administration of Fraction 1 tended to attenuate this weight loss. A glucose tolerance test revealed that mice treated with D-galactose exhibited elevated blood glucose levels at 30 min post-glucose loading (~135 mg/dL), which were significantly higher than those in both the control group and the D-galactose plus Fraction 1 group (Figure 2B). At 120 min, blood glucose levels in the Fraction 1-treated group decreased to 65 mg/dL, significantly lower than the 105 mg/dL observed in the D-galactose group. Area under the curve (AUC) analysis confirmed that Fraction 1 significantly improved glucose tolerance. Similarly, Hemoglobin A1c (HbA1c) levels were significantly increased in the D-galactose group, indicating prolonged hyperglycemia (Figure 2C), while this increase was significantly suppressed in mice receiving Fraction 1. To explore the mechanism underlying these effects, we evaluated blood insulin concentration and the homeostatic model assessment of insulin resistance (HOMA-IR) (Figure 2D). D-galactose administration significantly reduced insulin levels, while co-treatment with Fraction 1 preserved insulin secretion. HOMA-IR values did not differ significantly among the control, D-galactose, and Fraction 1 groups (Figure 2D), suggesting that the observed glucose intolerance was due to impaired insulin secretion rather than increased insulin resistance. These results demonstrate that D-galactose impairs pancreatic function, including β-cell insulin secretion, and that Fraction 1 exerts protective effects by maintaining insulin production and improving glucose tolerance.

### Effect of Fraction 1 on Pancreatic Tissue Aging Induced by D-Galactose Administration

**Figure 3:**
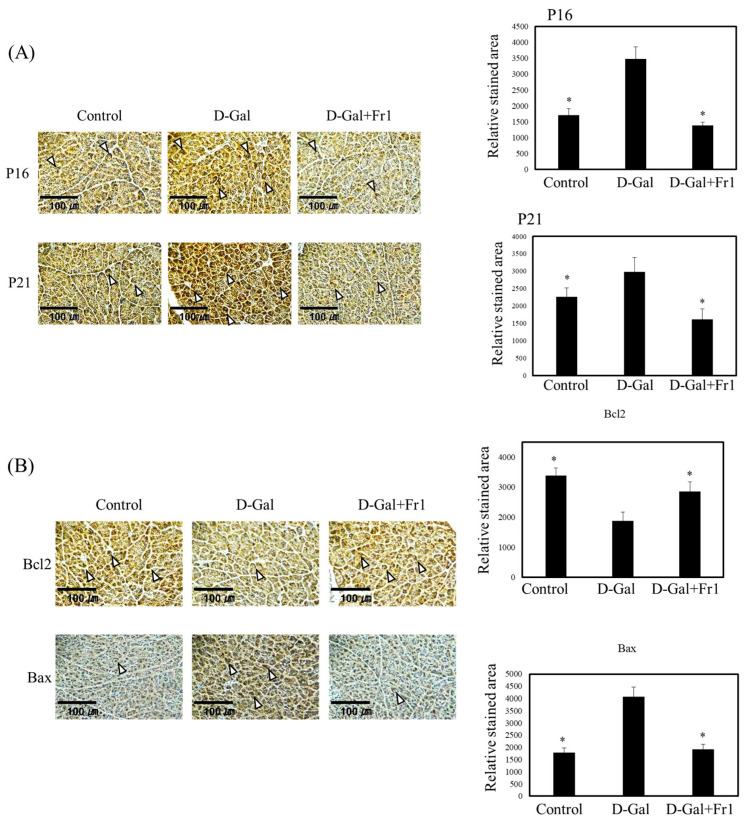

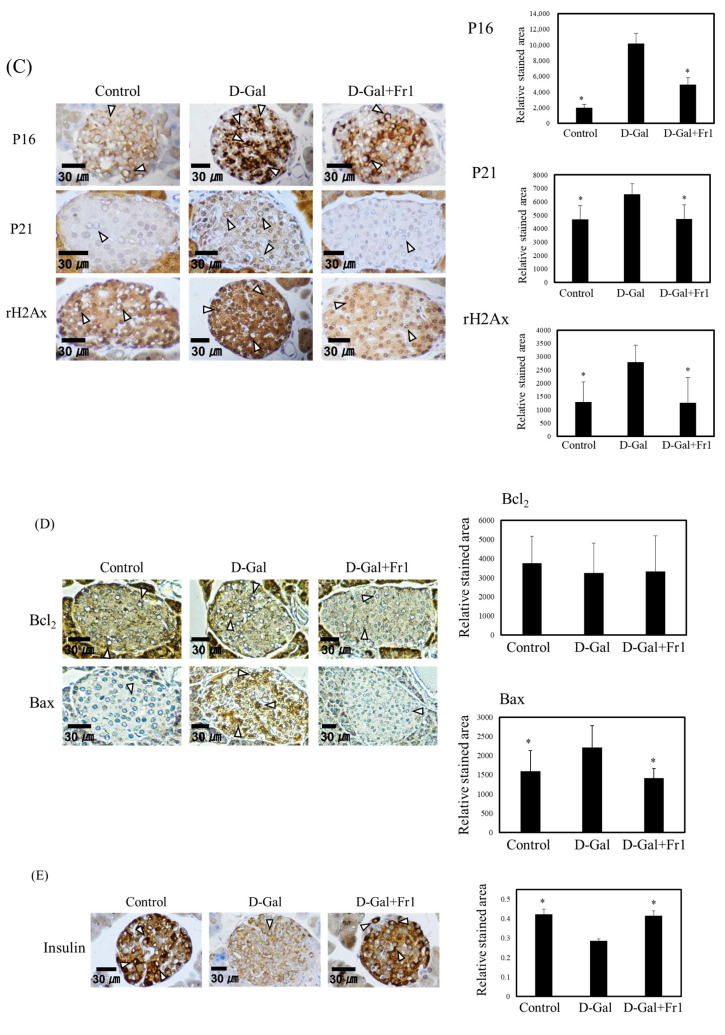
Effect of Fraction 1 on D-galactose-induced aging and apoptosis in pancreatic tissue. (**A**) Immunohistochemical staining for p16 and p21 in exocrine tissue. (**B**) Immunohistochemical staining for Bax and Bcl-2 in exocrine tissue. (**C**) Immunohistochemical staining for p16, p21, and γH2Ax in endocrine tissue. (**D**) Immunohistochemical staining for Bax and Bcl-2 in endocrine tissue. (**E**) Immunohistochemical staining for insulin in endocrine tissue. Scale bar represents 100 μm (**A**,**B**) or 30 μm (**C**,**D**). Arrowheads indicate stained areas. Data were collected from 10 to 20 randomly selected fields per section. Quantification of stained regions was performed using ImageJ (version 1.54). Data are presented as mean ± standard deviation. * *p* < 0.05 vs. D-galactose group.

To investigate whether the reduction in insulin secretion in the D-galactose group was attributable to pancreatic tissue damage or aging, histological and immunohistochemical analyses were performed. Immunostaining revealed that expression of the senescence markers p16 and p21 was significantly increased in the exocrine regions of the pancreas in the D-galactose group. In contrast, this upregulation was significantly attenuated in the Fraction 1-treated group (Figure 3A). Furthermore, D-galactose administration altered the expression of apoptosis-related proteins. Specifically, the pro-apoptotic protein Bax was upregulated, while the anti-apoptotic protein Bcl-2 was downregulated in the D-galactose group. These changes were substantially suppressed in the Fraction 1 group (Figure 3B). Within the endocrine region responsible for insulin production, the expression of p16 and p21 was also elevated in the D-galactose group but was significantly reduced following Fraction 1 treatment (Figure 3C). Similarly, γH2Ax, a DNA damage marker associated with cellular aging, was significantly upregulated in the D-galactose group and downregulated in the Fraction 1 group (Figure 3C). Bax expression in endocrine cells followed the same pattern, with significant elevation in the D-galactose group and suppression by Fraction 1 (Figure 3D), whereas Bcl-2 expression in the endocrine compartment remained largely unchanged. Immunostaining for insulin showed that D-galactose markedly decreased insulin expression in pancreatic endocrine cells, whereas this reduction was significantly prevented by co-administration of Fraction 1 (Figure 3E), suggesting that Fraction 1 preserves insulin synthesis at the tissue level.

These results indicate that D-galactose induces pancreatic dysfunction and impairs glucose metabolism through mechanisms involving increased cellular senescence, enhanced apoptosis, and decreased insulin production. Fraction 1 ameliorates these pathological changes by downregulating aging- and apoptosis-associated markers, preserving endocrine cell function, and maintaining insulin expression. Collectively, these findings suggest that the Fraction 1 isolated from nacre extract may serve as a promising therapeutic agent for preventing age-related pancreatic dysfunction and metabolic disorders.

## 3. Discussion

Pancreatic function progressively declines with age, resulting in impaired glucose tolerance and an increased risk of metabolic disorders, including type 2 diabetes [15,16,17]. D-galactose is widely used to model biological aging, as it induces oxidative stress and promotes inflammatory cytokine release [13,14]. In the present study, D-galactose administration induced metabolic disturbances resembling age-related dysfunction, such as elevated fasting blood glucose, impaired glucose tolerance, and increased HbA1c levels. Notably, co-administration of Fraction 1, a polysaccharide component isolated from nacre extract, significantly ameliorated these abnormalities, indicating a protective effect against D-galactose-induced metabolic aging. Although D-galactose is widely used as an experimental model of aging, direct evidence of its ability to reproduce pancreatic aging remains insufficient. Therefore, our results are more conservatively interpreted as demonstrating that the nacre-derived polysaccharide protects against D-galactose-induced pancreatic dysfunction, which may share features with aging-related changes.

Interestingly, although D-galactose markedly altered both glucose and insulin levels, HOMA-IR values remained unchanged among the groups. This finding suggests that glucose intolerance was primarily due to impaired insulin secretion rather than increased insulin resistance. This contrasts with the observations of Bo-Htay et al. [31], who reported elevated insulin resistance and increased insulin levels following subcutaneous D-glucose administration. Such discrepancies may reflect differences in administration route, dosage, treatment duration, and animal models. Consistent with the previous findings by El-Far et al. [32], histological and immunohistochemical analyses further revealed that D-galactose promotes cellular senescence and apoptosis in pancreatic tissue, as indicated by upregulation of p16, p21, and γH2AX, increased Bax, and reduced Bcl-2 expression. Importantly, Fraction 1 attenuated these alterations, thereby preserving the functional integrity of pancreatic endocrine cells and supporting insulin secretion.

Recent studies have demonstrated that various natural polysaccharides exert anti-aging and pancreas-protective effects. For example, polysaccharides derived from mulberry branches reduced fasting blood glucose and prevented pancreatic β-cell apoptosis in streptozotocin-induced diabetic mice by inhibiting pro-apoptotic signaling pathways [33]. Similarly, Zhang et al. showed that mushroom-derived polysaccharides from *Hericium erinaceus* improved glucose metabolism and enhanced antioxidant enzyme activities in diabetic mice [34]. In a zebrafish model, *Polygonatum sibiricum* polysaccharides were found to alleviate β-cell oxidative stress and apoptosis, thereby supporting insulin production [35]. These findings are consistent with our present results, highlighting the therapeutic potential of natural polysaccharides for the preservation of pancreatic function and prevention of age-related metabolic disorders.

Fraction 1 is a neutral polysaccharide predominantly composed of glucose, mannose, and rhamnose. This composition is entirely different from that of the sulfated polysaccharide previously isolated from nacre, which was rich in galactose and glucose [28]. Reports of other neutral polysaccharides with similar monosaccharide profiles support this finding. Reports of other neutral polysaccharides with similar monosaccharide profiles support this finding. For example, glucomannan-type polysaccharides from *Dendrobium officinale* improved glucose tolerance and preserved pancreatic β-cell function [36], while pumpkin polysaccharides containing more than 80% glucose demonstrated hypoglycemic and antioxidant activities in diabetic models [37]. Likewise, a rhamnose-enriched polysaccharide from *G. lithophila* enhanced insulin secretion and improved islet morphology in streptozotocin-induced diabetic rats [38]. Similarly, crude extracellular polysaccharides (EPS) derived from the edible mushroom Laetiporus sulphureus var. miniatus, which are rich in glucose, exerted a potent hypoglycemic effect in STZ-induced diabetic rats, accompanied by enhanced β-cell regeneration and upregulation of antioxidant enzymes [39]. Together, these studies indicate that rhamnose- and glucose-rich polysaccharides share common mechanisms such as improving glucose tolerance and alleviating hyperglycemia, consistent with the effects of Fraction 1 observed here.

While the exact molecular mechanisms require further investigation, our earlier data suggest that nacre extract possesses antioxidative properties in brain and skin [25]. It is plausible that similar antioxidative mechanisms protect pancreatic cells from D-galactose-induced senescence and apoptosis, thus maintaining tissue structure and function. Neutral polysaccharides may exert antioxidant activity via multiple mechanisms: scavenging free radicals through hydroxyl groups; chelating pro-oxidant metal ions; and enhancing endogenous antioxidant enzymes such as superoxide dismutase, catalase, and glutathione peroxidase, as demonstrated both in reviews and experimental studies [40,41,42].

A limitation of the present study is the absence of a pharmacological positive control, such as metformin or rapamycin [43], which are widely used as reference agents in metabolic and aging-related studies. In this study, we focused solely on elucidating the intrinsic effects of the nacre-derived polysaccharide fraction without direct comparison to standard drugs. Future investigations incorporating such positive controls will be essential to further validate and contextualize the therapeutic potential of nacre polysaccharides. In addition, although we confirmed that Fraction 1 is a neutral polysaccharide with negligible protein contamination—as SDS-PAGE with silver staining showed no detectable protein bands, and mass spectrometry provided results consistent with a polysaccharide composition—its detailed structural characteristics, including glycosidic linkages and higher-order conformations, remain to be clarified. To address this limitation, we plan to conduct further investigations such as NMR spectroscopy and methylation analysis.

In conclusion, our findings demonstrate that the neutral polysaccharide fraction (Fraction 1) derived from nacre extract protects against D-galactose-induced pancreatic and metabolic dysfunction. By supporting insulin secretion, suppressing cellular senescence, and attenuating apoptosis, Fraction 1 contributes to the maintenance of glucose tolerance and metabolic health. These results provide a promising foundation for the development of nacre-derived polysaccharides as natural therapeutic candidates for age-related metabolic diseases.

## 4. Materials and Methods

### 4.1. Materials

Pearl oyster shells (Pinctada maxima) were collected from Iki Bay, Nagasaki, Japan. Antibodies against Bcl-2, Bax, p21, insulin, γ-H2AX, and cyclin-dependent kinase inhibitor 2A (p16) were purchased from Biorbyt (San Francisco, CA, USA).

### 4.2. Preparation and Fractionation of Nacre Extract

Nacre extract was prepared as previously described [26,27], with minor modifications. The prismatic layer of the shells was removed, and the nacreous layer was ground into a fine powder. The powdered nacre was decalcified using 10% acetic acid, followed by dialysis (molecular weight cutoff: 10,000 Da) against deionized water. The dialyzed extract was lyophilized and re-extracted with deionized water.

The resulting water-soluble fraction was initially subjected to reverse-phase chromatography using a C18 column (Tosoh, Tokyo, Japan) and eluted with a linear gradient of acetonitrile (0–50%). Each collected fraction was administered intraperitoneally to D-galactose-treated mice (4–5 mice per group), and fractions that effectively suppressed the elevation of fasting blood glucose levels were identified.

The most active fraction was further purified by anion-exchange chromatography using a TSKgel DEAE-5PW column (Tosoh, Tokyo, Japan) with a linear NaCl gradient (0–0.5 M) in 20 mM Tris-HCl buffer (pH 7.5). The bioactive fractions were pooled, dialyzed using a 3000 Da molecular weight cut-off membrane to remove low-molecular-weight compounds, and lyophilized. The resulting neutral polysaccharide fraction was designated as Fraction 1 and used in subsequent experiments. To evaluate its purity and composition, SDS-PAGE followed by silver staining was performed to assess protein contamination, monosaccharide composition was determined and MALDI-TOF mass spectrometry was carried out for molecular characterization. A total of 100 g of powdered nacreous layer yielded approximately 0.3 mg of Fraction 1, corresponding to 0.0003% of the starting material.

### 4.3. Chemical Composition and Structural Characterization of Fraction 1

The carbohydrate and protein contents of Fraction 1 were determined by the phenol–sulfuric acid and BCA assay, respectively.

#### 4.3.1. FT-IR ATR Spectroscopy

Fourier-transform infrared (FT-IR) spectra of the polysaccharide sample were recorded over a range of 4000–400 cm^−1^ using an FT/IR-4000 spectrometer (JASCO, Tokyo, Japan) equipped with an attenuated total reflectance (ATR) accessory to identify functional groups.

#### 4.3.2. MALDI-TOF MS Analysis

MALDI-TOF mass spectrometry was performed using an UltrafleXtreme™ mass spectrometer (Bruker Daltonics, Bremen, Germany). Fraction 1 was mixed with 2,5-dihydroxybenzoic acid (DHB) as the matrix and spotted onto the MALDI plate for analysis. 

#### 4.3.3. Monosaccharide Analysis

Monosaccharide composition was determined by 4-aminobenzoic acid ethyl ester (ABEE) labeling followed by high-performance liquid chromatography (HPLC), as previously described [26]. Briefly, Fraction 1 (1 mg) was hydrolyzed in 2 M trifluoroacetic acid (TFA) at 100 °C for 4 h. The hydrolysate was dried under reduced pressure, washed with methanol, and reconstituted in distilled water.

For ABEE labeling, 10 μL of the hydrolysate was mixed with 10 μL of ABEE solution (0.35 M in acetic acid/methanol, 3:7, *v*/*v*) and 10 μL of sodium borate buffer (0.5 M, pH 8.5). The mixture was incubated at 80 °C for 1 h, then evaporated to dryness. The labeled monosaccharides were extracted with chloroform and water, and the aqueous phase was analyzed by HPLC (Shimadzu, Kyoto, Japan) using a reverse-phase column (Shim-pack VP-ODS, 4.6 × 150 mm) with detection at 254 nm. Monosaccharides were identified and quantified by comparison with ABEE-labeled standards.

### 4.4. Animals

Male ICR mice (4 weeks old) were obtained from CLEA Japan (Tokyo, Japan). Mice were housed under controlled conditions (24 °C, 50% humidity, 12 h light/dark cycle) with ad libitum access to AIN-76A diet and water. All mice were acclimated for one week prior to experimentation. D-galactose was administered intraperitoneally at a dose of 500 mg/kg/day for eight weeks in both the D-galactose and D-galactose + nacre fraction-treated groups. Control mice received PBS injections. For screening bioactive fractions, each chromatographic fraction from nacre extract was administered intraperitoneally. The dose of each fraction was calculated based on the amount obtained from the active dose of the nacre extract. Fraction 1, the most active fraction, was subsequently administered at 20 mg/kg/day for eight weeks to the D-galactose + Fraction 1 group. Each group consisted of 5–6 mice. Animals were randomly assigned to each treatment group. Sample size was determined based on prior studies using similar D-galactose models [25]. A priori power analysis was not conducted, and no predefined inclusion or exclusion criteria were applied. All animals were included in the final analysis. At the end of treatment, mice underwent glucose tolerance testing and were euthanized. Pancreatic tissues were rapidly harvested, snap-frozen in liquid nitrogen, and stored at −80 °C. All procedures followed guidelines approved by the Animal Ethics Committee of the Muroran Institute of Technology on February 3, 2022 (approval number: H30KS01), and were conducted in accordance with ethical guidelines to minimize animal suffering. Animals were monitored daily for general health status. No adverse events were observed during the study, and no specific humane endpoints were established.

### 4.5. Immunohistochemistry

Paraffin-embedded pancreatic sections were deparaffinized in xylene, rehydrated in graded ethanol, and subjected to antigen retrieval in citrate buffer (pH 6.0) at 95 °C for 20 min. Endogenous peroxidase activity was blocked with 3% hydrogen peroxide at room temperature for 10 min. Sections were incubated overnight at 4 °C with primary antibodies, followed by polymer-based detection using a one-step IHC kit (VitroVivo Biotech, MD, USA). DAB was used as the chromogen, and sections were counterstained with hematoxylin. Immunoreactive areas were quantified using ImageJ (NIH, USA). Images were converted to 8-bit grayscale, and a fixed threshold was applied to measure DAB-stained areas. Data were collected from 10 to 20 randomly selected fields per section and averaged for analysis.

### 4.6. Blood Biochemistry

The primary outcome measure was fasting blood glucose level. Blood glucose was measured from tail vein samples using a glucometer (FreeStyle Lite, Abbott, ON, Canada). Serum insulin levels were determined by ELISA (Biomatik, Wilmington, DE, USA). Insulin resistance was assessed using the homeostatic model assessment (HOMA-IR):

HOMA-IR = [Fasting insulin (μU/mL) × Fasting glucose (mg/dL)]/405

For glucose tolerance testing, mice were fasted for 24 h and then given glucose orally (2 g/kg). Blood glucose was measured at 0, 30, 60, 120, and 180 min post-administration. Each time point was measured in triplicate, and AUC was calculated over 0–180 min. HbA1c levels were measured using a mouse-specific ELISA kit (Biomatik).

### 4.7. Statistical Analysis

All data are presented as mean ± standard deviation (SD) from five or six mice per group. Statistical significance was assessed by one-way ANOVA followed by Dunnett’s post hoc test, using BellCurve for Excel software (version 2.15; Tokyo, Japan). A *p*-value < 0.05 was considered statistically significant. All experiments were independently repeated at least twice to confirm reproducibility. Outcome assessors were blinded to group allocation during data analysis. Assumptions of normality and homogeneity of variance were considered but were not formally tested.

## 5. Conclusions

This study demonstrates that neutral polysaccharide (Fraction 1) isolated from nacre extract effectively protects against D-galactose-induced pancreatic dysfunction and glucose metabolism dysfunction in mice. Fraction 1 improved glucose tolerance, preserved insulin secretion, and suppressed cellular senescence and apoptosis in pancreatic tissue. These findings support the potential of nacre-derived polysaccharides as natural therapeutic agents for age-related metabolic disorders.

## Figures and Tables

**Table 1:** Monosaccharide composition in Fraction 1.

Monosaccharide	nmol
GlcA	0
GalA	0
Gal	5.8
Man	52.1
Glc	84.8
Ara	0
Rib	0
Xyl	9.5
ManNAc	0
GlcNAc	1.2
Rha	66.1
Fuc	2
GalNAc	11.7
Total	233.3

Abbreviation: GlcA: Glucuronic acid, GalA: Galacturonic acid, Gal: Galactose, Man: Mannose, Glc: Glucose, Ara: Arabinose, Rib: Ribose, Xyl: Xylose, ManNAc: N-acetyl-D-mannosamine, GlcNAc: N-acetylglucosamine, Rha: Rhamnose, Fuc: Fucose, GalNAc: N-Acetylgalactosamine.

## Data Availability

The data used during the current study are available from the corresponding author on reasonable request.

## Supplementary Materials

The following supporting information can be downloaded at: https://www.mdpi.com/article/10.3390/molecules30173555/s1, Figure S1: SDS-PAGE analysis of the F1 fraction. After electrophoresis, the gel was silver-stained. MW denotes molecular weight markers (kDa).

## Abbreviations

The following abbreviations are used in this manuscript:

DAB	3,3’-diaminobenzidine
FT-IR	Fourier-transform infrared
ATR	Attenuated total reflectance
MALDI-TOF-MS	Matrix-assisted laser desorption/ionization time-of-flight mass spectrometry
ELISA	Enzyme-linked immunosorbent assay
HOMA-IR	Homeostatic model assessment of insulin resistance
AUC	Area under the curve
HbA1c	Hemoglobin A1c
ABEE	4-aminobenzoic acid ethyl ester
