# Supplementary figures and images for: Protective Effect of a Highly Enriched Nacre-Derived Neutral Polysaccharide Fraction on D-Galactose-Induced Pancreatic Dysfunction
Source: Molecules. 2025 Aug 30;30(17):3555. doi: 10.3390/molecules30173555 (PMC12430628; doi:10.3390/molecules30173555)

## Slide 1
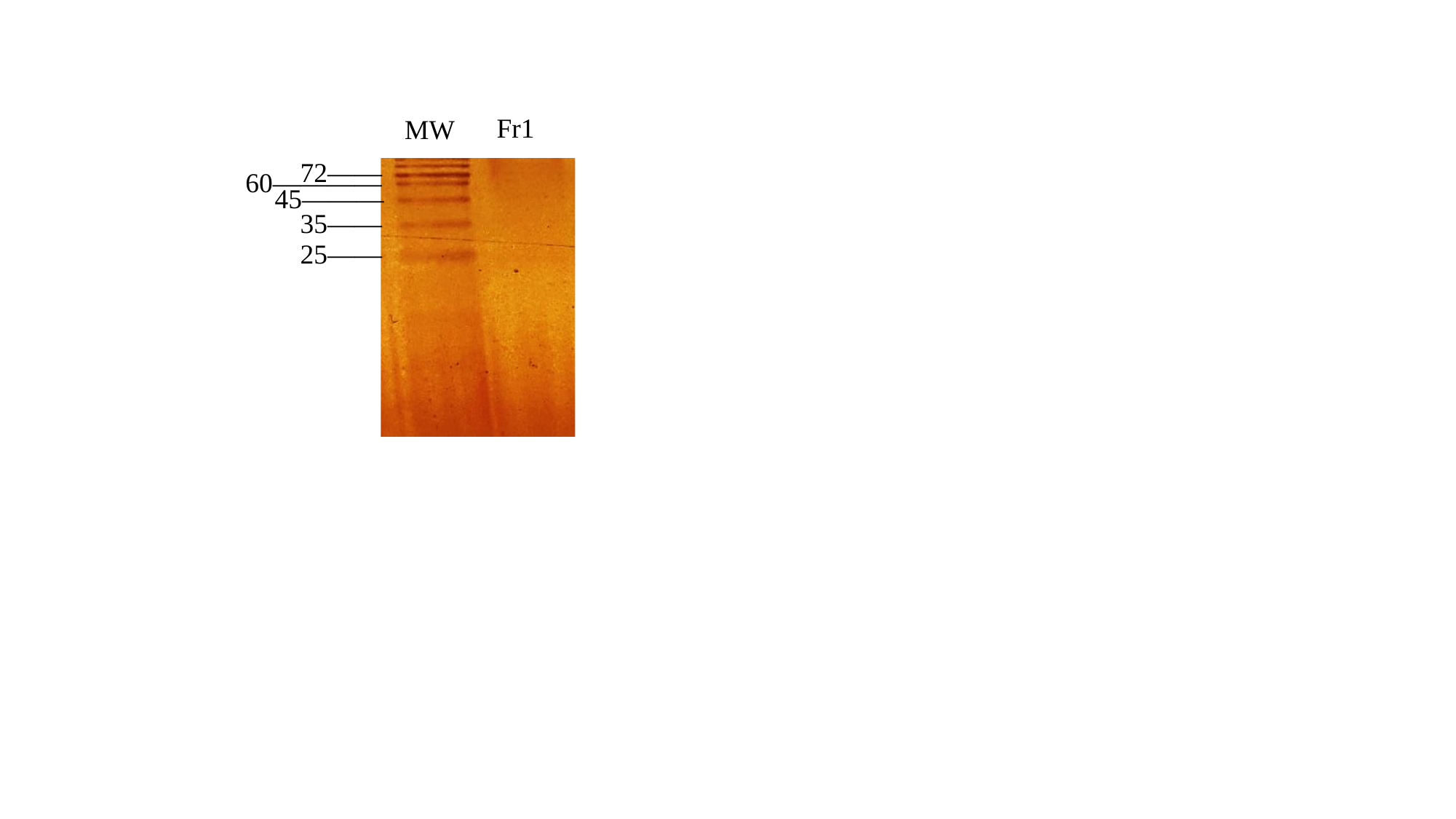

Fr1
MW
72——
60————
45———
35——
25——

Supplement: Supplementary file 1 [file molecules-30-03555-s001.zip › molecules-3816111-supplementary.pptx]
